# HSQC-NMR spectroscopy and exploratory data analysis of crude oil residue in relation to the time of spill

**DOI:** 10.1039/d5ra00826c

**Published:** 2025-06-04

**Authors:** N. D. Menkiti, C. Isanbor, O. O. Ayejuyo

**Affiliations:** a Department of Chemistry, Ahmadu Bello University Zaria Nigeria mcmenkiti007@gmail.com; b Department of Chemistry, University of Lagos Akoka Nigeria

## Abstract

Eight oil residues extracted from crude oil spill sites have been investigated for natural attenuation using heteronuclear single quantum coherence nuclear magnetic resonance spectroscopy (2D HSQC NMR). Using the exploratory data analysis (EDA) techniques; principal component analysis (PCA) and hierarchical cluster analysis (HCA), the predictive ability of the NMR technique with respect to similarities and differences in the composition of the oil residue over time was explored. The first three PCs from PCA accounted for 87% of the total variance while three clusters each were obtained from HCA analysis based on similarity in samples and NMR areas. Both exploratory analyses revealed that the –CH_3_/–CH_2_ types, aliphatic, and aromatic content of the oil residue are the main factors responsible for compositional differences. The Euclidean distance constructed from PCA indicated real differences between fresh crude oil, aged, and younger residue. If the exposure time of the oil spill is known, HSQC coupled with exploratory data analysis would be a useful tool in evaluating the structural and compositional transformation of oil residue in the environment. This may be useful as a guide in deciding which remediation strategy is implemented in an oil spill environment like the Niger Delta region.

## Abstract 2

EDA techniques such as PCA and HCA were used to investigate NMR spectroscopy's ability to predict changes in oil residue composition, identifying –CH_3_/–CH_2_ types and aliphatic/aromatic content as key factors driving these variations.

## Graphical abstract

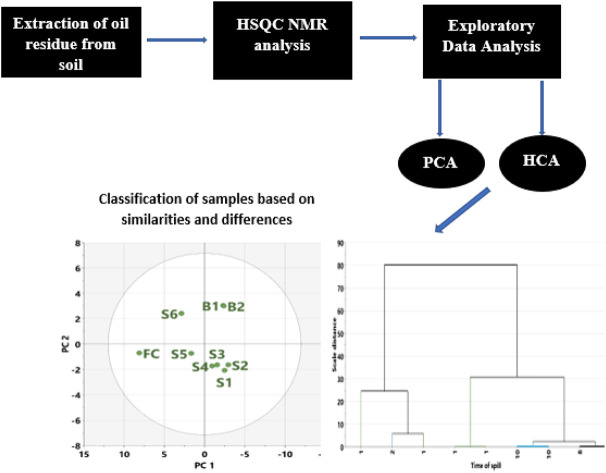

## 1. Introduction

Crude oil, otherwise known as petroleum, contains a lot of substances belonging to homologous series of hydrocarbons (aliphatic, alicyclic and aromatic hydrocarbon) as well as heteroatomic compounds (resins and asphaltenes) containing heteroatoms such as sulfur, oxygen and nitrogen and a very little amount of trace metals (V, Ni, Fe, Zn, *etc.*).^1–3^ When crude oil spills on land, it undergoes composition and structural changes due to environmental exposure conditions. Processes like photooxidation, biodegradation, and adsorption are responsible for these changes.^4^ In the soil, aerobic biodegradation of aromatic hydrocarbon has led to the formation of new compounds.^5,6^ It is important to state that these new compounds formed are site-specific and composition will differ due to microorganisms, pathways of degradation, and the time of the spill. What this means is that each oil spill will present a unique composition and hence, the toxicity of its components will vary.^7,8^ Treatment of crude oil-contaminated sites requires a fundamental understanding of the composition and physical properties of the oil residue. Various methods have been used to reclaim the soil *viz.*; bioremediation,^9,10^ biopile,^11^ natural attenuation,^12^ and soil washing using solvents and surfactants.^13–15^ The success of the remediation method applied depends on qualitative and quantitative information derived from studies on the fate and behaviour of crude oil contaminants in the environment. Investigations into the composition and structural changes of oil residue in the environment have been done in the past using both physical and chemical techniques. This includes gas chromatography, quantitative fluorescence, IR spectroscopy, and HPLC among others.^16,17^ These methods of analysis have their shortcomings. For instance, GC and GC-MS may require derivatization, and HPLC requires some other sample preparation procedures,^18^ or some special columns to resolve aromatic and resin fractions. For UV spectroscopic analysis, asphaltene content will aggregate at high concentrations of crude oil making noise evident in the spectrum,^19^ or too low a concentration may exclude the detection of some species.^20^ Quantitative fluorescence spectroscopy can only provide characteristics of crude oil based on certain chromatographic variables,^21^ and on the assumption that weathered crude oil and fresh crude oil have similar properties if it is to be applied for source identification.^22^ In contrast to these techniques, nuclear magnetic resonance spectroscopy (NMR) allows for both qualitative and quantitative descriptions of a mixture of complex organic compounds especially crude oil.^23,24^ (NMR spectroscopy that offers a rapid characterization of the oil residue properties is required since oil spill occurs regularly.^25^ It gives molecular functional group information about the properties of samples at the macroscopic scale, and this is what allows for suitable characterization.^26–28^ In recent times, NMR spectroscopy has been directed at improving the sensitivity and resolution of the technique. It is for this reason that 2D NMR (COSY, TOCSY, NOESY, HQSC, HMBC experiment) spectroscopy is at the forefront of complex mixture elucidation. For these systems, two-dimensional heteronuclear shift correlated methods give sufficient discrimination of resonance, as the correlated peaks are separated by chemical shifts of two nuclei,^29^ and have shown themselves particularly well in determining the chemical and spatial structure.^30,31^ It has been shown that NMR data are useful in evaluating the average molecular properties of crude oil,^32–35^ as well as the overall transformational changes in oil residue in soil.^36,37^ In soil systems, the structural changes are difficult to reproduce in the laboratory, hence, measurements of field samples are of fundamental interest. The Niger Delta environment in Nigeria provides an excellent opportunity to monitor and characterize the weathering processes in the soil under natural conditions that ultimately leads to the variation in the chemical composition of the spilled oil over time. Together with established supervised and unsupervised machine learning tools using multivariate methods such as principal component analysis, PCA,^38–41^ and hierarchical cluster analysis, HCA,^42^ or orthogonal projections to latent structures, OPLS,^43^ this approach can be used to identify metabolites that vary between different classes of samples. Recently, we showed that similarities and differences in the structural composition of aged oil residue samples from the Niger Delta area of Nigeria can be identified using ^1^H-NMR with PCA.^44^ Unlike other techniques such as GC-MS, GC × GC-MS, or HPLC, NMR does not resolve the components in a sample. Hence, the compositional similarity and differences embedded in the oil residue can be extracted using multivariate analysis. To the best of our knowledge, there are not yet any combined methods of 2D HSQC NMR and exploratory data analysis in relation to the time of spill that has been used to elucidate compositional changes in the oil spills in the natural environment of the Niger Delta in Nigeria. In this work, further analysis of the oil residue was carried out to determine if predefined NMR parameters obtained from the 2D HSQC NMR experiment and exploratory data analysis could satisfactorily evaluate the compositional changes of oil residue in terms of similarities and differences in relation to the time of the spill.

## 2. Materials and methods

### 2.1 Sampling and sample properties

Soil samples, S1–S6 were obtained from crude oil contaminated sites in the Niger Delta region with a history of spillage. The oil spills were of different ages, crude oil sources and environmental exposure conditions. Samples B1 and B2 were obtained by the process of biostimulation by using cow dung as fertilizer to improve the degradation of the fresh crude oil in a controlled environment,^45^ while FC is a fresh crude oil sample that we assumed had not undergone significant degradation. Some properties of the soil, oil residue, and fresh crude oil were determined and have been described elsewhere.^46^

### 2.2 Data acquisition and processing

The Heteronuclear Single Quantum Correlation (HSQC) NMR spectra of the fresh crude oil (FC) and the extract of eight oil residues (S1–B2) were recorded by a Bruker AC 500 MHz spectrometer. Approximately 5 mg of the crude oil mixture was accurately weighed and dissolved in 500 μL of CDCl_3_ (Aldrich, 99.8%) containing 0.03% tetramethyl silane (TMS) as an internal standard. The solution was mixed thoroughly into a 5 mm NMR tube to ensure complete dissolution. The sample concentration was thus approximately 10 mg mL^−1^ (10 000 mg L^−1^), consistent with typical recommendations for HSQC experiments of complex mixtures. This concentration was selected to ensure an adequate signal-to-noise ratio, particularly for low-abundance functional groups, and to avoid potential issues with weak solvent residual peaks and insufficient cross-peak intensity in the HSQC spectra.^42^ HSQC experiment was carried out using echo/anti-echo-time proportional phase incrementation gradient selection with decoupling during acquisition.^47–49^ All spectra were automatically corrected for phase and baseline. The total runtime of the experiment was about 1 hour and 30 minutes for each sample. The integrated signal volumes of selected cross-peaks in the HSQC spectra were obtained using the integral function of the software, with consistent integration parameters applied across all samples. To account for possible intensity variations arising from differences in heteronuclear coupling constants (^1^JCH) and relaxation times among functional groups, all HSQC spectra were normalized to total spectral area before chemometric analysis. The signal volumes and their assignment used for the study are given in Table 1.

**Table 1:** Assignment in the HSQC spectra of the samples^11^

Area	*δ*_H_ (ppm)		*δ*_C_ (ppm)		Assignment
	From	To	From	To	
X1	0.79	0.93	10.02	12.56	–CH_3_ β to CH (ethyl group)
X2	0.74	1.03	12.56	15.9	–CH_3_ γ or > to fg^a^ (aromatic ring) or γ to
X3	0.74	1.00	18.12	21.52	–CH
X4	0.76	1.00	21.58	24.02	–CH_3_ α to –CH and β or > to –CH_2_
X5	0.91	0.98	26.74	29.06	–CH_3_ α to –CH and to –CH
Q1	1.25	1.33	12.94	15.68	–CH_3_ α to quaternary C
Q2	1.22	1.36	19.23	21.32	–CH_3_ β to an aromatic ring
Q3	1.21	1.37	21.38	23.77	–CH_2_ α to –CH_3_ and β to CH
Q4	1.16	1.36	23.78	25.65	–CH_2_ α to –CH_3_ and γ or > to –CH
Q5	1.15	1.37	25.74	28.1	–CH_2_ β to quaternary C
Q6	1.02	1.48	28.1	31.15	–CH_2_ β to –CH
Q7	1.19	1.31	31.23	32.71	–CH_2_ γ or > to –CH_3_, –CH or another fg
Q8	1.31	1.43	31.81	33.79	–CH_2_ β to –CH_3_ and γ or > to CH_3_, –CH or another fg
Q9	1.27	1.35	34.01	34.99	–CH in cyclohexane with –CH β
Q10	1.19	1.34	35.73	38.64	–CH in cyclohexane
Q11	1.1	1.19	38.62	40.52	–CH_2_ α to –CH and β to –CH_3_
A1	1.59	1.76	25.43	27.98	–CH_2_ α to quaternary C with 2(–CH_3_)
A2	1.48	1.57	27.05	28.83	–CH_2_ β to aromatic ring and β to –CH
A3	1.56	1.71	34.43	38.04	–CH α to 2(–CH_3_) and –CH_2_
Y1	2.09	2.92	17.66	22.95	–CH_2_ in cycloalkane α to –CH
Y2	2.52	2.96	27.52	32.14	–CH_3_ α to aromatic rings
Y3	2.41	2.63	31.82	37.58	–CH_2_ α to aromatic ring and α to –CH_3_ (substituted ethylene) or –CH_2_ in cyclohexane α to an aromatic ring
C	6.74	7.9	121.87	132.33	–CH_2_ α to aromatic ring and γ or > to
					–CH_3_, –CH or another fg
					–CH in aromatic ring

a fg – functional group.

### 2.3 Exploratory data analysis

Principal Component Analysis (PCA) and Hierarchical Clustering Analysis (HCA) were applied to the 2D-HSQC NMR spectra data to reveal the underlying dataset structure, making it possible to identify similarities between samples and detect potential outliers. The different areas from the HSQC NMR experiment represent variables that will determine the relationship between samples that have undergone the same type of structural changes through time even though environmental conditions will not be the same. The software Originpro2019b was used to perform the PCA and HCA analysis and visualized using SIMCA 17(Umetrics).

#### 2.3.1 Principal component analysis (PCA)

Principal component analysis (PCA) and other data reduction methods for spectroscopic study are straightforward as they help visualize the most important information contained in a data set by taking multiple variables and reducing them into a much smaller and more manageable number of variables while retaining most of the information the original variables contained.^50^ Mathematically, PCA is based on a decomposition of the covariance matrix of the variables in a data set. Given a data matrix **X** with *m* rows of samples and *n* columns of variables, the covariance matrix of **X** is defined in eqn (1) as:1
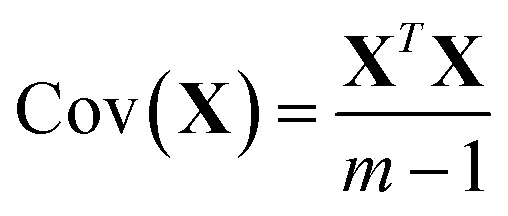


The result of the PCA procedure is a decomposition of the data matrix **X** (eqn (2)) into principal components called score and loading vectors. Here ***t***_**1**_, is the score vector, ***p***_**1**_ is the loading vector, and **E** is the residual matrix.

2 **X**_nxm_ = ***t***_**1**_***p***_**1**_^*T*^ + ***t***_**2**_***p***_**2**_^*T*^ + ***t***_***i***_***p***_***i***_^*T*^⋯ + ***t***_***k***_***p***_***k***_^*T*^ + **E**_nxm_

The score and loading vectors contain information on how the samples and variables, respectively relate to each other. The direction of the first principal component (***t***_**1**_, ***p***_**1**_) is the line in the variable space that best describes the variation in the data matrix **X**. The direction of the second principal component is given by the straight line that best describes the variation not described by the first principal component and so on. Thus, the original data set can be adequately described using a few orthogonal principal components instead of the original variables, with no significant loss of information.

#### 2.3.2 Hierarchical clustering analysis

Hierarchical cluster analysis (HCA) is a pattern recognition tool that reveals the inherent relationship between variables or subjects without any prior assumption.^51^ As a multivariate statistical technique, HCA resolves and groups variables based on the properties they possess.^52^ The resulting groups which are now called ‘clusters’ exhibit high internal (within clusters) homogeneity and high external (between groups) heterogeneity and the level of similarities at which observations are merged is used to construct a dendrogram.^53,54^ For continuous variables, like our data, the Euclidean distance is the best choice for the distance metric, because interpoint distances between the samples can be computed directly by eqn (3);3
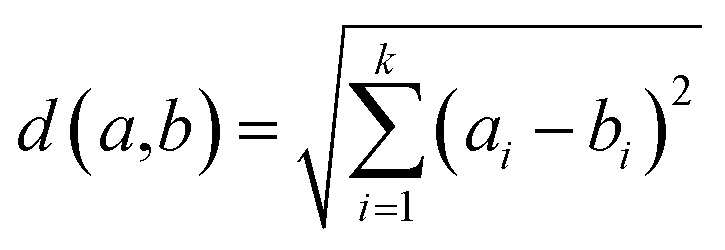


HCA seeks to form natural object grouping based exclusively on similarities in predictor variables. The similarity between each pair, *a* and *b*, of crude oil NMR responses was computed as the Euclidian distance: where *k* is the number of NMR variables. After that, clusters *A* and *B*, formed by samples in close proximity, were grouped into a binary hierarchical tree (or dendrogram) in which the distance between clusters *D* (*A*, *B*) is the longest distance between two samples, *a* Ꞓ *A* and *b* Ꞓ *B*. A cut-off distance was applied to the resulting dendrogram to obtain an arbitrary number of clusters.^42^ For cluster analysis, it is best to autoscale the data, because similarity is directly determined by a majority vote of the measurement variables.

## 3. Results and discussions

### 3.1 Sample characteristics

The choice of sampling was based on a site with a history of oil spills of a known time. The sample label and time of spill are given in Table 2.

**Table 2:** Sample code and time of spill

Sample	S1	S2	S3	S4	S5	S6	B1	B2	FC
Time of spill (years)	10	10	8	5	2	1	1	1	1
Designation	Older residue			Younger residue					

**Fig. 1:**
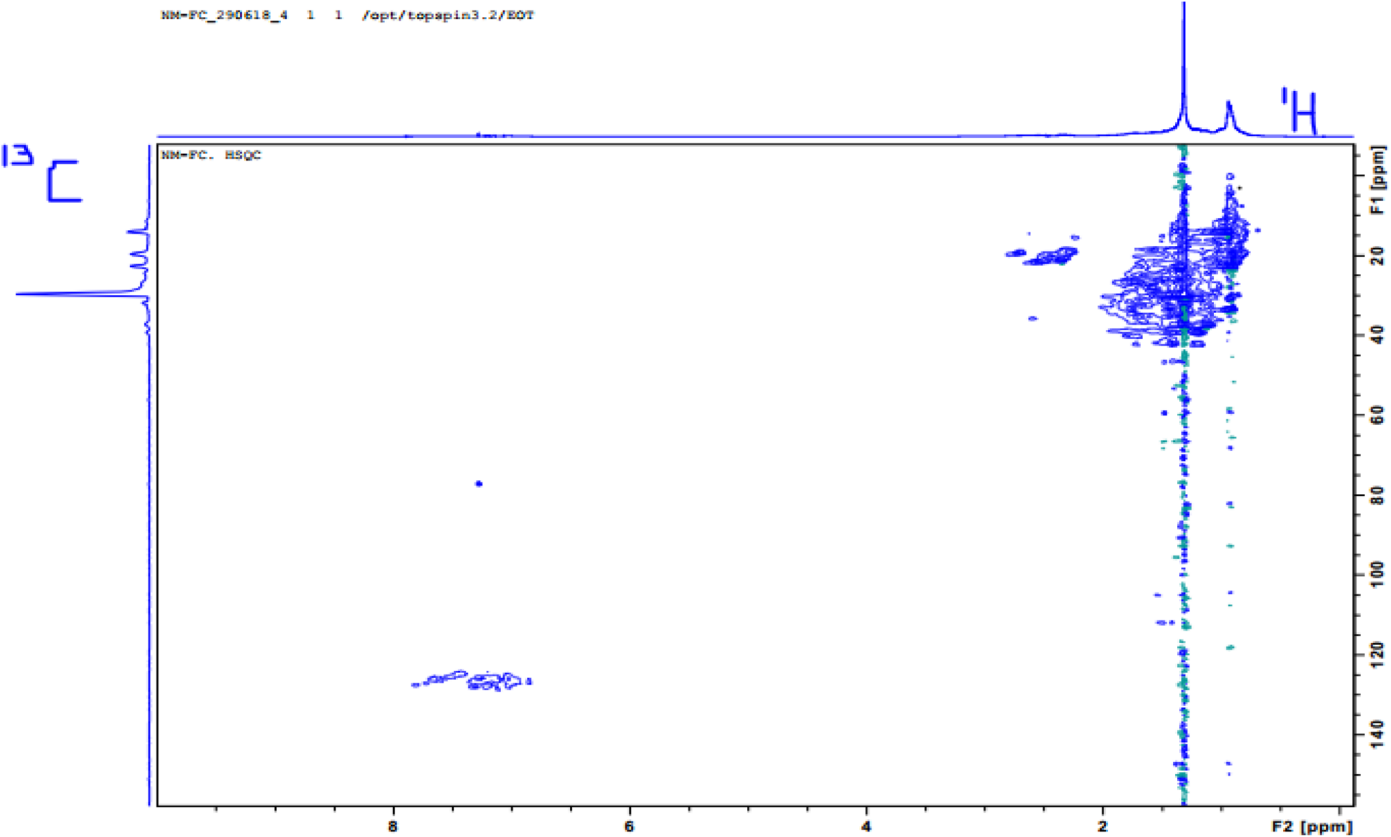
^1^H, ^13^C-HSQC (500 MHz) NMR spectrum of fresh crude oil (FC) sample in CDCl_3_.

**Fig. 2:**
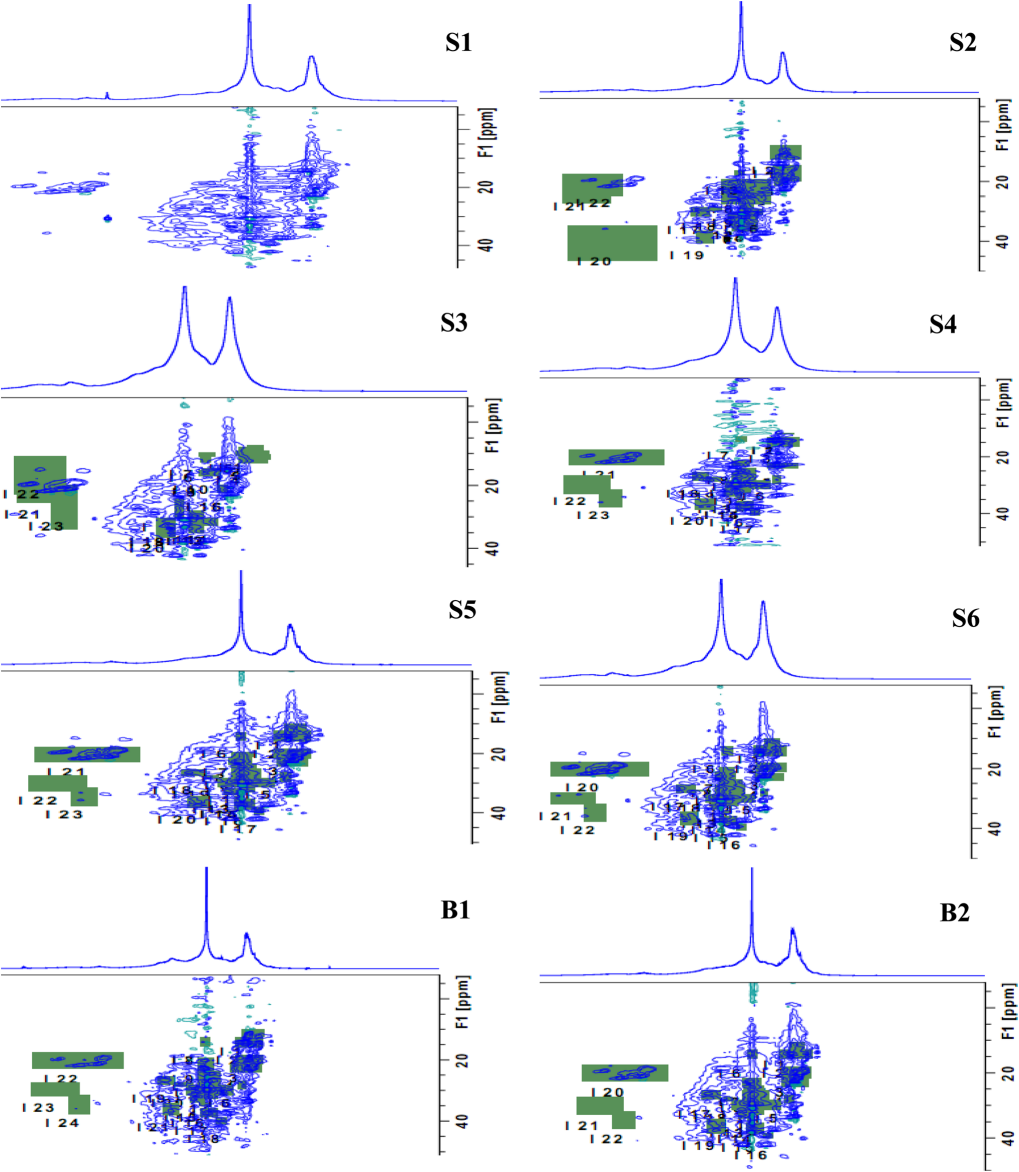
Fingerprint of the expanded HSQC-NMR spectra (*δ*_C_/*δ*_H_ 10–45/0.3–3 ppm) for oil residue; green areas indicate integrated region and sample S1 was left out for clarity.

Fig. 1 and 2 show the HSQC spectrum of fresh crude oil and the integrated signal volumes obtained from oil residue samples, respectively. From Fig. 1, two groups of signals are observed: aliphatic area of proton (^1^H, 1–4 ppm), carbon (^13^C, 10–60 ppm), and aromatic areas; ^1^H (6–8 ppm), ^13^C (115–145 ppm). According to Marshall and Rodgers,^55^ crude oil contains tens of thousands or hundreds of thousands of compounds, hence, is arguably the world's most compositionally complex organic mixture, therefore, it is expected that the HSQC NMR spectra should show a crowded and overlapping profile, as seen in Fig. 2. These spectra can be described as a superposition of the hydrogen and carbon chemical shifts of each pure constituent, weighted by the concentration of the constituent. It is expected that the usual oil residue will contain the same hydrocarbon families of saturates, aromatics, resins, and asphaltenes in various proportions as environmental exposure will not be the same across the study area. It is in this light that differences and similarities are expected between the HSQC NMR profile (confined within a relatively narrow spectral window) of the oil residue. Though there is an overlap of signals, we rely on statistical analysis to make the differences in oil residue samples obvious (Table 3).

**Table 3:** Integrated signal volumes of selected cross-peaks in HSQC spectra

Name	S1	S2	S3	S4	S5	S6	B1	B2	FC
X1	48.48	46.35	50.322	51.13	61.038	67.36	4.2923	9.99	101.87
X2	130.02	173.47	193.47	183.3	191.45	199.38	135.046	307.19	291.64
X3	255.93	248.35	268.43	287.3	224.89	237.02	63.35	79.33	354.32
X4	218.74	110.03	210.86	226.7	491.42	427.98	52.75	94.97	522.12
X5	1.99	3.95	3.95	4.14	12.05	16.66	6.477	9.837	22.98
Q1	24.64	23.91	33.91	33.22	74.08	78.09	48.32	44.78	100.95
Q2	37.37	33.17	39.17	48.72	142.56	198.12	171.67	173.15	242.31
Q3	82.67	83.11	83.11	85.33	101.12	164.01	154.26	153.53	101.12
Q4	226.21	117.43	159.3	190.53	488.52	401.35	9.5839	26.45	873.57
Q5	127.76	168.87	189.41	199.54	213.27	297.02	314.82	307.33	320.32
Q6	554.51	648.79	756.92	890.09	458.86	983	749.52	798.34	1034.68
Q7	40.09	34.2	49.76	52.43	45.17	72.71	79.59	22.678	95.62
Q8	109.07	107.22	152.09	152.61	193.631	191.38	216.28	112.75	391.12
Q9	16.85	10.31	15.31	20.22	60.55	29.16	8.6	9.7	90.42
Q10	81.01	89.79	106.47	109.22	316.29	381.18	30.89	22.61	423.28
Q11	30.38	22.41	29.5	33.25	31.21	24.29	28.07	27.783	62.44
A1	53.35	39.09	45.67	63.11	210.02	211.19	72.11	78.104	245.01
A2	30.72	20.58	29.58	31.24	32.37	25.44	26.06	29.25	68.44
A3	75.4	63.48	93.48	74.35	179.64	199.74	92.93	86.67	265.45
Y1	77.34	85.65	95.42	99.66	11.31	535.09	117.26	114.23	21.27
Y2	30.08	28.44	31.44	54.34	10.44	61.22	9.9363	9.59	60.23
Y3	129.51	176.98	196.09	201.22	151.64	58.71	12.22	15.003	250.43
C	400.55	352.1	456.33	512.26	754.12	854.43	229.27	220.72	987.46

### 3.2 Principal component analysis

The properties of the soil have been described elsewhere with percentage SARA composition in the following order: saturates > aromatics > polar > asphaltene.^46^ In our previous ^1^H-NMR measurement, the spectrum was divided into regions with structural groups assigned based on the proton environment.^44^ However, HSQC NMR offers a unique and better advantage of producing well-resolved parts and has successfully been applied to complex petroleum fractions,^28^ coal-derived fuel, shale oil,^56^ and biopiles.^11^

**Fig. 3:**
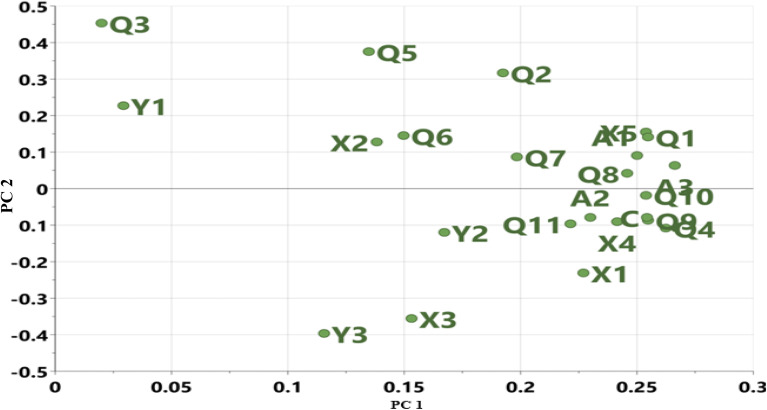
Loading plot of HSQC NMR signal assignment in Table 1.

Table 4 shows the cumulative component matrix and the component factors after rotation. These results allow for the grouping of initial variables (HSQC areas) according to the strength of the connection with the principal components. This study also used the loading diagram of PC 1 and PC 2 (Fig. 3) to visualize which HSQC NMR weathering indices provide similar information, which ones are negatively correlated or not related to each other, and which ones are not well explained by the model (PC 1 and PC 2 close to 0). In Table 4, HSQC areas that show a moderate to high correlation are highlighted. The three PCs selected accounted for over 87% of the total variance. In this manner, the areas X2, X5-Q2, Q5-Q9, and Q11-A3 are associated with PC 1 (48%). Also, X1, X3, X4, Q4, and B2-C were loaded on PC 2 (33%), while Q3 and X1 are associated with PC 3 (12%). A2 and X2 are related to alkyl-substituted aromatic compounds and –CH_2_ β to an aromatic ring. PC 1 has positive factor loading values and is highly influenced by X2 (–CH_3_ γ to an aromatic ring), X5 (–CH_3_ α to –CH and –CH_3_), Q1 (–CH_3_ β to an aromatic ring), and Q5-Q9 (–CH_2_ or –CH in alicyclic compounds). PC 2 is influenced by Y2 (–CH_2_ α to an aromatic ring), Y3 (–CH_2_ α to an aromatic ring or γ to another functional group), C (H-aromatics), and lastly, by X4 and Q10, which are indicators of branched alkanes. Such classes of hydrocarbons are present in younger residue than in aged oil that has undergone extensive weathering. PC 3 was influenced by Q3 (–CH_2_ α to CH_3_) and D1 (–CH_3_ α to aromatic ring). In the PCA analysis, we did not carry out any prior pre-classification, so samples with similar attenuation processes are expected to be close to each other. Samples S2, S3, B1, and B2 have a positive PC 1 that is characterized by X1, X3, X4, and Y2 which are –CH_3_ of saturated hydrocarbons, alkenes, and –CH_3_ β to CH. Also, samples S5 and S6 are positively loaded on PC 2 and are characterized by X2, Q2, Q3, Q5, Q6, Q7, Q8, Q10, A1 and A3, which suggest the influence of α and β-alkyl fragments of naphthenic and hydroaromatic compounds.

**Table 4:** Loading of variables on PCs

Variable	Principal component		
	PC 1	PC 2	PC 3
X1	0.287	**0.943**	−0.117
X2	**0.666**	−0.014	−0.149
X3	−0.075	**0.952**	−0.245
X4	0.506	**0.766**	0.020
X5	**0.898**	0.371	0.154
Q1	**0.892**	0.384	0.128
Q2	**0.963**	−0.053	0.248
Q3	0.527	−0.503	**0.683**
Q4	0.620	**0.746**	−0.176
Q5	**0.876**	−0.274	0.315
Q6	**0.501**	−0.257	0.364
Q7	**0.625**	0.383	0.165
Q8	**0.826**	0.405	−0.169
Q9	**0.698**	0.606	−0.316
Q10	0.591	**0.739**	0.183
Q11	**0.668**	0.449	−0.494
A1	**0.775**	0.497	0.168
A2	**0.706**	0.454	−0.457
A3	**0.806**	0.550	0.095
Y1	0.036	0.142	**0.980**
Y2	0.129	**0.799**	0.297
Y3	−0.107	**0.772**	−0.561
C	0.519	**0.826**	0.114
Eigenvalue	13.269	4.730	2.120
% variance	40.86	33.64	12.96
% cumm variance	40.46	74.51	87.47

**Fig. 4:**
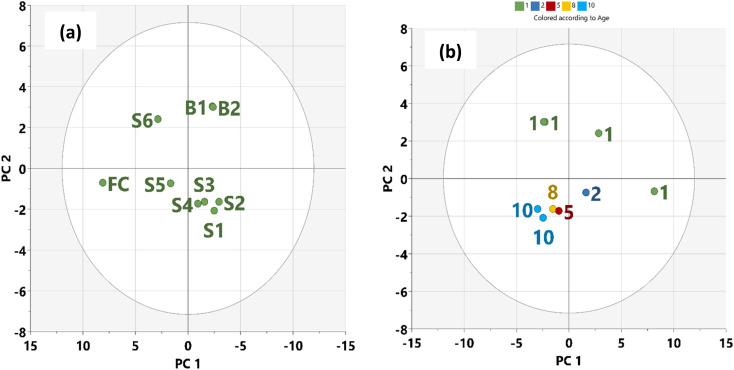
Score plot: (a) oil residues (b) time of spill.

Based on the score plot (Fig. 4a), the naturally attenuated older residue samples (S1, S2, S3) are closer together, as are the younger residues (S5 and S6) closer to FC. Remarkably, samples B1 and B2 will show marked differences in degradation because microbial action on the oil within one year is close to each other in the score plot. Mansurova *et al.* (2024)^57^ have obtained similar results and interpretations. Fig. 4b shows the score plot of the time evolution of the samples with HSQC NMR parameters. In this spatial time distribution, the degraded oil residues evolved in a clockwise pattern. In addition, cluster formation in PCA plots (Fig. 4b) revealed similarities among data points within respective samples, corresponding to assigned oil spill categories and demonstrating statistical differentiation between these oil residues.^58^ This observation allows for the predictability of the time of spill of crude oil when NMR indices are known for such samples.

We applied the Euclidean distance (Table 5) based on the similarity of the samples to gain insight into their compositional relationship so that it will be consistent with the time of the spill. On this basis, the average distance (0.8) between FC and the naturally attenuated younger residue, S5 and S6 was lower compared to the average distance (1.4) between FC and older residue S1–S3 (Table 5, in bold). Biostimulated samples B1 and B2 had the largest Euclidean distance (2.8). This shows that the natural attenuation in the Niger Delta region within the period under study may not be more effective than biostimulation. Therefore, more time may be needed if transformational changes due to natural attenuation will be significant.

**Table 5:** Euclidean distance between FC and oil residue samples

Sample	S1	S2	S3	S4	S5	S6	B1	B2
Distance	**1.2**	**1.7**	**1.5**	1.5	0.7	0.9	**2.8**	**2.8**

### 3.3 Hierarchical cluster analysis

**Fig. 5:**
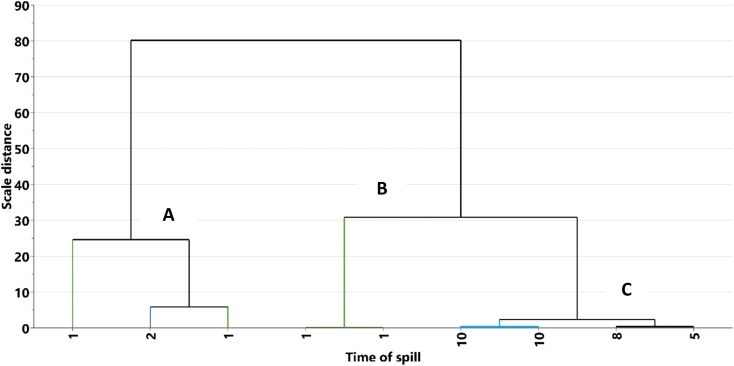
Dendrograms from hierarchical clustering of the oil residue in the soil at different time of spill.

**Fig. 6:**
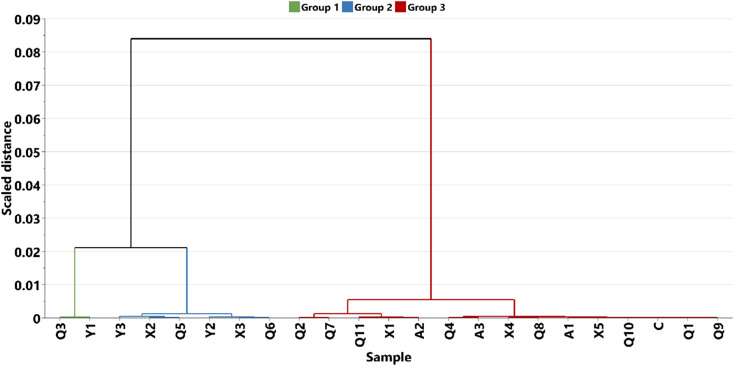
Dendrograms from hierarchical clustering of the oil residue in the soil at based on HQSC NMR weathering index.

In this study, HCA was used to arrange the eight (8) oil residue samples and the 23 HSQC-NMR areas into dendrogram based on their similarities. Where samples are similar, the dendrogram offers a visual indication by bringing them close to each other in a cluster. Fig. 5 and 6 show the dendrogram for the time of the spill and HSQC-NMR areas, respectively. Based on the individual sample characteristics, three clusters were obtained and labelled as A, B, and C. It was observed that cluster A, contained oil residue (S4–S6) with a time of spill of less than five years while cluster B, had samples that were biostimulated (B1, B2). Cluster C contained older residues (S1–S3). This agrees with our previous report,^45^ and related work by Rios *et al.*^11^ Similarly, in the dendrogram built from the NMR areas (Fig. 6), three clusters were identified. Group 1 contains arene, protonated arenes, and internal aromatic carbon atoms.^57^ Group 2 contains –CH_3_ and –CH_2_ of aromatic and other functional groups. These functional groups are due to alcoholic and carboxylic polar compounds formed from biodegradation.^44,58^ Lastly, group 3 contains γ-CH_2_, –CH_2_, and –CH of aliphatic and naphthalene groups. This classification agrees well with our previous study on where the structural differences lie in the samples. A comparison of the two dendrograms, as in Fig. 5 and 6, showed that the main factor responsible for grouping is aliphatic and aromatic composition. However, we must stress some level of ambiguity in this classification as in the case of S4 (<5 years) which was grouped with older residue.

## 4. Conclusion

The analysis of naturally attenuated oil residues using data from specific areas of HSQC NMR spectroscopy and exploratory data analysis (PCA and HCA) have shown that differences in environmental evolution in samples are characterized by differences in –CH_3_/–CH_2_, aliphatic and aromatic content of the oil residues. The Euclidean distance constructed from PCA showed the real difference between fresh crude oil, aged, and younger residue. If the time of the spill is known, HSQC NMR would be a useful tool in evaluating the structural and compositional transformation of oil residue in the environment.

## Data availability

The data supporting the findings of this study are all in the original manuscript.

## Author contribution

Nnamdi Menkiti: methodology, formal analysis, investigation & writing the original draft. Chukwuemeka Isanbor: conceptualization, supervision, review & editing. Olusegun Ayejuyo: supervision, review & editing.

## Conflicts of interest

The authors declare that they have no known competing financial interests or personal relationships that could have appeared to influence the work reported in this paper.
